# The effects of daily fasting hours on shaping gut microbiota in mice

**DOI:** 10.1186/s12866-020-01754-2

**Published:** 2020-03-24

**Authors:** Linghao Li, Yuxin Su, Fanglin Li, Yueying Wang, Zhongren Ma, Zhuo Li, Junhong Su

**Affiliations:** 1 China-Malaysia National Joint Laboratory, Biomedical Research Center, Northwest Minzu University, Lanzhou, China; 2 Department of Basic Medicine, Medical School, Kunming University of Science and Technology, No.727 South Jingming Rd., Chenggong District, Kunming, China; 3 Department of Gastroenterology and Hepatology, Erasmus MC-University Medical Center, Rotterdam, The Netherlands

**Keywords:** Daily fasting hours, Gut microbiota, Food intake, Mouse model

## Abstract

**Background:**

It has recently been reported that intermittent fasting shapes the gut microbiota to benefit health, but this effect may be influenced to the exact fasting protocols. The purpose of this study was to assess the effects of different daily fasting hours on shaping the gut microbiota in mice. Healthy C57BL/6 J male mice were subjected to 12, 16 or 20 h fasting per day for 1 month, and then fed ad libitum for an extended month. Gut microbiota was analyzed by 16S rRNA gene-based sequencing and food intake was recorded as well.

**Results:**

We found that cumulative food intake was not changed in the group with 12 h daily fasting, but significantly decreased in the 16 and 20 h fasting groups. The composition of gut microbiota was altered by all these types of intermittent fasting. At genus level, 16 h fasting led to increased level of *Akkermansia* and decreased level of *Alistipes*, but these effects disappeared after the cessation of fasting. No taxonomic differences were identified in the other two groups.

**Conclusions:**

These data indicated that intermittent fasting shapes gut microbiota in healthy mice, and the length of daily fasting interval may influence the outcome of intermittent fasting.

## Background

Gut microbiota consists of a group of microorganisms that live in the mammalian intestinal tract and plays key roles in health and disease. Gut microbiota is not only involved in a number of major physiological processes including fermentation of indigestible dietary polysaccharides and synthesis of essential amino acids and vitamins, but also is a vital factor in maintaining gut homeostasis for host [1]. However, a normal gut microbiota can be negatively affected by multiple environmental and host genetic factors and thus is converted into a dysbiotic state [2]. Due to various public health problems like metabolic syndrome and cancer that are associated with the dysbiosis of gut microbiota [2], restoring or promoting a healthy microbiota has been therefore regarded as one of promising approaches for the prevention and treatment of these health problems [3].

Intermittent fasting as an emerging dieting concept is usually practiced by restricting eating from 12 to 24 h (hrs). A great number of studies have provided evidence for health benefits of intermittent fasting to host [4–6]. The strategies of intermittent fasting can differ dramatically, for instance according to different daily hours of fasting. A well-known intermittent fasting pattern is Ramadan fasting, which entails abstinence from eating and drinking from sunrise to sunset over a period of approximately 30 days during the month of Ramadan [7], and is being widely studied for its impact on human health and disease in population-based studies [8–10]. Another popular fasting pattern is every other day fasting, which has been shown to improve obesity and multiple sclerosis in experimental model through restoring gut microbiota [11, 12]. These studies indicate the promising potential of intermittent fasting in shaping gut microbiota. Obviously, daily fasting hours is varied across different studies, ranging between 12 to 24 h. However, how the length of daily fasting hours affect the outcome of fasting and gut microbiota still remains largely unclear so far.

To shed a light on daily fasting hours on gut microbiota, this study investigated the effects of 12, 16, and 20 h daily fasting for 1 month on gut microbiota in mice. By profiling fecal bacterial community with 16S rRNA gene sequencing, we found that intermittent fasting altered the gut microbiota, and the effect was more robust in mice treated with daily 16 h fasting.

## Results

### Different fasting protocols on cumulative food intake

**Fig. 1:**
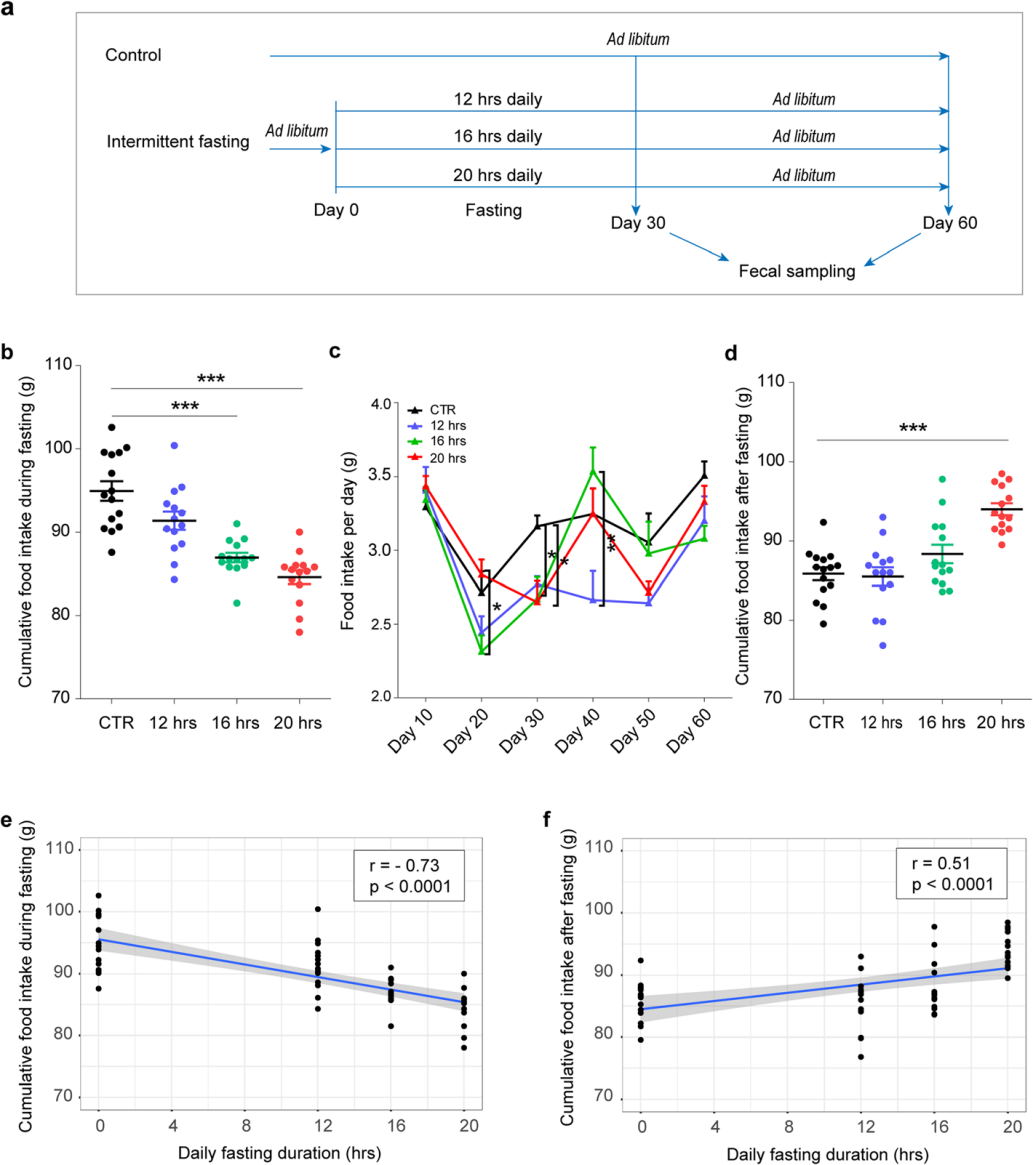
Food intake during intermittent fasting. **a** Experimental design; **b** The amount of total food intake during fasting; **c** The amount of food intake per day at each time point during the two-month study period; **d** The amount of total food intake 1 month after the cessation of intermittent fasting. *p < 0.05, **p < 0.01, ***p < 0.001 by one-way ANOVA, followed by Tukey’s post hoc for multiple comparisons. **e** Pearson correlation between cumulative food intake and daily fasting hours during fasting. **f** Pearson correlation between cumulative food intake and daily fasting hours 1 month after the cessation of fasting

C57BL/6 J mice were divided into three groups on the basis of daily fasting duration, as shown in Fig. 1a. Mice fed Ad libitum were used as control (CTR). Food intake was measured for the indicated period of time. We found that cumulative food intake during the period of fasting (30 days) was significantly reduced in the 16 and 20 h fasting groups compared to CTR (*p* < 0.0001), but was not changed in the 12 h group (Fig. 1b). Also, daily food intake was changed similarly during fasting (Fig. 1c; *p* < 0.05). One month after the cessation of intermittent fasting, in comparison to CTR, cumulative food intake was significantly increased in the 20 h fasting group but not in the 12 h and 16 h groups (Fig. 1d). Additionally, a very strong negative correlation (*r* = − 0.73, *p* < 0.0001) was found between the length of daily fasting time and cumulative food intake during fasting (Fig. 1e), but the two variables, in turn, were positively correlated (*r* = 0.51, *p* < 0.0001) after the cessation of fasting (Fig. 1f). These findings support prior research indicating that mice underwent a period of fasting learned quickly that food would not be continuously available and thus tended to gorge [13, 14].

**Fig. 2:**
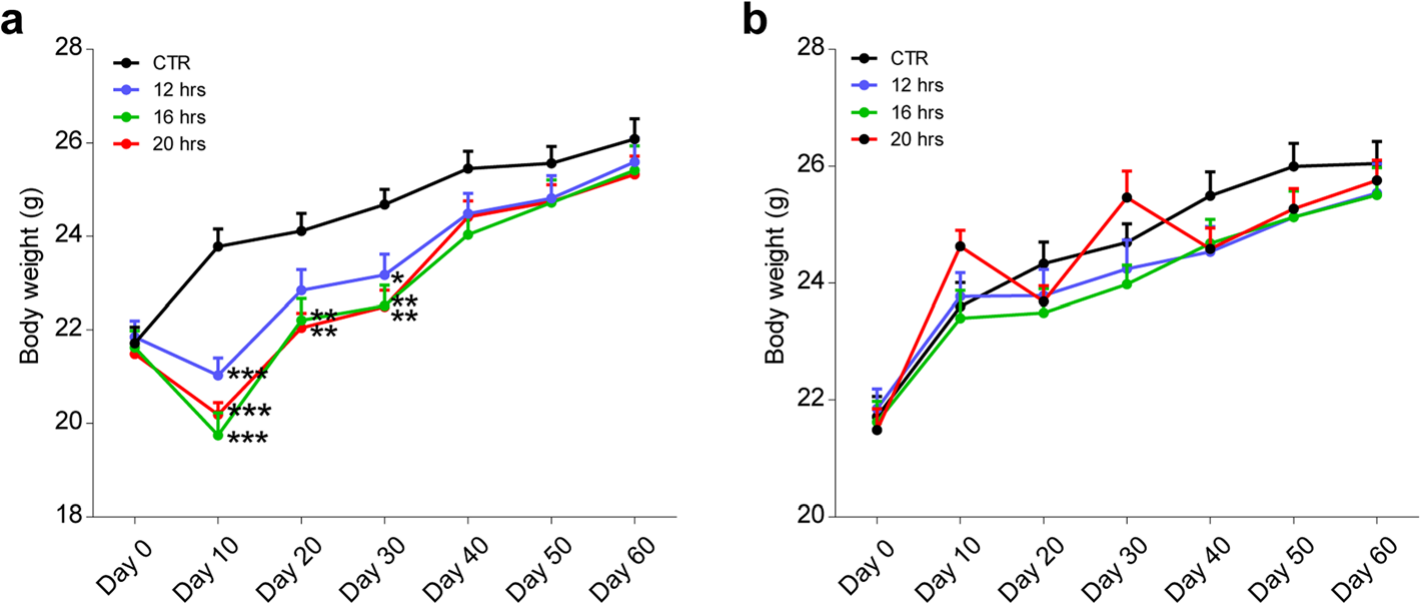
Changes in body weight during intermittent fasting. **a** Body weight at the end of daily fasting; **b** Body weight before the start of daily fasting; *p < 0.05, **p < 0.01, ***p < 0.001 by one-way ANOVA, followed by Tukey’s post hoc for multiple comparisons

Although body weight measured before eating was reduced in all groups (Fig. 2a), the difference was disappeared immediately after eating (Fig. 2b), suggesting that body weight was relatively stable for healthy C57BL/6 J mice under these conditions. Given the fact that a fairly large reduction in food intake (Fig. 1b) did not result in reduction of body weight after the limited feeding period (Fig. 2b), which might be due to a shorter period of intermittent fasting.

### The effects of daily fasting hours on gut microbiota

To study the effect of different fasting regimens in changing gut microbiota in healthy mice, fecal microbial communities were analyzed using 16S rRNA approach. After filtering and length trimming, a total number of high-quality sequences generated from all fecal samples was 7,509,548. The average number of the reads per sample was 66,456 ± 6127 (mean ± SD). Next, we calculated values of alpha diversity. There were no differences in alpha diversity between CTR and any of the fasting groups (Table 1).

**Table 1:** Microbiome alpha diversity indices between different time durations at the end of fasting or 1 month after the cessation of intermittent fasting

Time point	Indices	Time duration			
		CTR	12 hrs	16 hrs	20 hrs
Day 30	Shannon	5.269 (0.871)	5.092 (0.806)	5.487 (0.529)	4.680 (0.868)
	Simpson	0.909 (0.107)	0.897 (0.070)	0.934 (0.040)	0.879 (0.073)
Day 60	Shannon	5.406 (0.802)	5.160 (0.918)	5.363 (0.737)	4.850 (1.406)
	Simpson	0.917 (0.065)	0.905 (0.088)	0.925 (0.054)	0.851 (0.174)

*Abbreviation*: *CTR* Control or no fasting

Alpha diversity was calculated for both richness and evenness by the Shannon diversity index and Simpson index. Differences in Shannon and Simpson index values were determined by one-way ANOVA, followed by Tukey’s post hoc test for multiple comparisons. Data are presented as mean and standard deviation (S.D.)

**Fig. 3:**
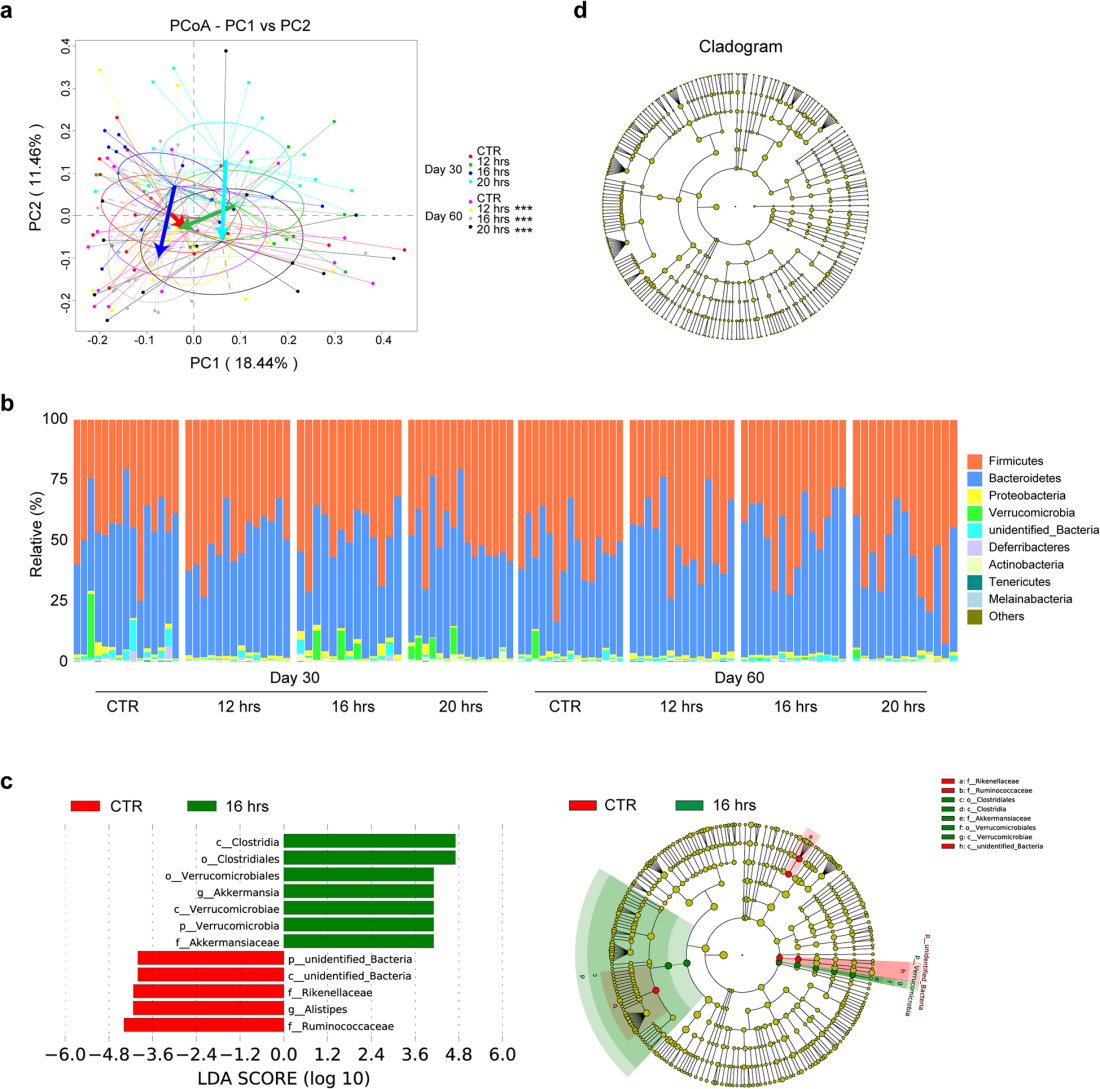
Analysis of gut bacterial communities by 16S rRNA analysis from fed and fasted mice. **a** Principal Co-ordinates Analysis (PCoA) on Bray-Curtis dissimilarities of bacterial communities from four different fasting regimens at two time points. Each point corresponds to a community from a single mouse. Colors indicate community identity. Ellipses show the 95% confidence intervals. Coloured arrows indicate community shift from day 30 to day 60. Intra-group differences were indicated by using ANOSIM test. ***p ≤ 0.001. **b** The Figure shows the percentage of each community contributed by the indicated phyla. Time point and daily fasting durations are indicated below the Figure. Taxa that discriminated between fasted and control mice during fasting (**c**) or 1 month after the cessation of fasting (**d**). Taxa with a log LDA (linear discriminant analysis) score above 4.00 as determined by using LEfSe. Data shown are the log10 linear discriminant analysis (LDA) scores following LEfSe analyses and the hierarch of discriminating taxa visualized as cladograms for taxonomic comparisons between fasted and control mice

To assess the changes in microbiota composition after fasting, analysis of similarities (ANOSIM) and a multivariate ANOVA based on similarity tests (Adonis) using Bray-Curtis distance matrix were performed with 999 permutations of the data. As shown in Table 2, significant changes in gut microbiota composition were observed between fasting and non-fasting mice, but also among subgroups by duration of daily fasting (Table 2). However, no statistically significant difference was observed between any groups after the cessation of fasting, except for 16 h regimen (Table 2). This might be related to the difference in feeding rate after discontinuation of fasting (Fig. 1c). To further illustrate bacterial community changes after fasting, principal coordinates analysis (PCoA) was performed using Bray-Curtis distances (Fig. 3a). As expected, significant changes were observed in all fasting groups; no difference was found in CTR (Fig. 3a).

**Table 2:** Analysis of beta diversity of gut microbiota by ANOSIM and Adonis test

Time point	Method	Comparison					
		CTR-12 hrs	CTR- 16 hrs	CTR- 20 hrs	12 hrs–16 hrs	12 hrs–20 hrs	16 hrs–20 hrs
Day 30	**ANOSIM**						
	*R* value	0.207	0.168	0.202	0.119	0.075	0.094
	*p* value	0.005**	0.003**	0.002**	0.027*	0.062	0.039*
	**Adonis**						
	*R*^2^ value	0.099	0.095	0.099	0.081	0.066	0.077
	*p* value	0.002**	0.001***	0.007**	0.036*	0.038*	0.026*
Day 60	**ANOSIM**						
	*R* value	0.023	0.111	0.027	0.002	0.012	0.082
	*p* value	0.230	0.014*	0.236	0.406	0.34	0.062
	**Adonis**						
	*R*^2^ value	0.047	0.066	0.042	0.042	0.042	0.061
	*p* value	0.171	0.030*	0.298	0.304	0.316	0.050

Analysis of similarity was calculated between durations of daily fasting for 1 month based on OTUs tables of Bray-Curtis distance matrices. Each pairwise comparison of two groups was performed using 999 permutations. ∗*p* < 0.05, ∗∗*p* < 0.01, ∗∗∗*p* ≤ 0.001

At the phylum level, mouse gut microbiota in the present study was largely enriched in *Firmicutes* and *Bacteroidetes* (Fig. 3b), which are similar to previous studies [15, 16]. To further identify microbiota taxa that account for the greatest differences between fasted and unfasted mice, we performed Liner Discriminate Analysis (LDA) coupled with effect size measurements (LEfSe). As shown in Fig. 3c, multiple taxonomic differences were found between 16 h daily fasting and CTR; pneumotype was enriched with operational taxonomic units (OTUs) from the class Clostridia to the family Akkermansiaceae, and was reduced with the class unidentified_Bacteria and families including Rikenellaceae and Ruminococcaceae. At the genus level, a genus with higher abundance was *Akkermansia*, whereas *Alistipes* from family Rikenellaceae was reduced (Fig. 3c). However, these effects disappeared 1 month after the cessation of fasting (Fig. 3d). It should be noted that one mouse in CTR group has the highest relative abundance of *Akkermansia* (Additional file 2: Figure S1). Reasons for this are unknown, but individual variation might play a role in this regard. No taxonomic differences were found between CTR and the other two groups during fasting. These data suggest that the length of daily fasting time should be considered when intermittent fasting is used as a strategy for interventions for shaping gut microbiota.

## Discussion

Fasting, in particular intermittent and timing fasting, is being widely practiced for various purposes in global population, for at least millennia now. Recently, intermittent fasting is gaining scientific interest as a potential intervention to improve health [17, 18]. In this study, we hypothesized that gut microbiome is shaped to different extents by different daily fasting duration over a period of 30 days and our results are in line with this notion.

The duration of fasting has important effects on physiological and metabolic processes in human and animal models [19, 20]. A recent study has reported that daily fasting (13 h) improves health and survival in mice independent of diet composition and calories [14]. However, the study did not investigate changes in gut microbiota during fasting. In our study, we found that cumulative food intake was also not significantly affected in mice treated with 12 h daily fasting, but the composition of their gut microbiota was altered. Our findings are consistent with a recent study conducted in obese mice that cumulative food intake was not affected by every other day fasting either, but has gut microbiota altered [21].

The gut microbiome is becoming increasingly recognized as an important host genome and plays a substantial role in maintaining physiological homeostasis [2, 22]. In turn, the resulting dysbiosis of the gut microbiota is highly associated with the pathogenesis of both acute or chronic diseases, in particular digestive disorders including inflammatory bowel disease, liver cirrhosis and colorectal cancer, and thus is responsible for the unrelenting increase in so-called diseases-of-affluence [23]. In this study, we have demonstrated that intermittent fasting for 30 days, even with 12 h daily fasting duration, is sufficient to alter the composition of gut microbiota. The results support the notion that fasting may become as an alternative strategy for more effective restoration of human gut microbiota composition.

The health benefits of intermittent fasting on aging, antioxidant stress, metabolism and cardiovascular disease have been demonstrated in human and animal studies [18, 24–27], but its effect on gut microbiota remains largely unclear. In our study, we found 30 days of daily fasting (16 h fasting) led to significantly increased level of *Akkermansia* and deceased level of *Alistipes*. Importantly, previous studies have demonstrated that increase in *Akkermansia spp*. is associated with metabolic improvements including decreased liver triglyceride accumulation and alleviated intestinal inflammation [28], and reduction in *Alistipes* might also improve intestinal inflammation [29, 30]. Taken together, these findings suggest that the beneficial effect of intermittent fasting on health is likely to be linked to gut microbiota alterations during fasting, with particular reference to increase in specific species frequently reported to be anti-inflammatory species and reduction in species often described as pro-inflammatory.

A remaining question of interest, however, is whether the readout of intermittent fasting under longer daily duration is due to fasting duration, reduction of food intake or both. This is also in favor of the mechanistic distinction between intermittent fasting and classical caloric restriction.

## Conclusions

We have demonstrated that intermittent fasting shapes the gut microbiota in healthy mice in a daily fasting hour dependent manner, and a magnitude effect of fasting was observed upon a 16 h fasting duration. However, these effects gradually disappear when the fasting is discontinued. It must also be borne in mind that this study was only conducted in a small group of animals over a short period of time. Future research is hence needed to determine an optimal fasting regimen(s) that can provide long term beneficial effect.

## Methods

### Animal and study design

Sixty male specific pathogen-free C57BL/6JLvri mice (6-wk-old, initial weight 18–20 g) were purchased from Lanzhou Veterinary Research Institute of Chinese academy of agricultural sciences. Animals were housed 5 per polypropylene cage on sterilized wood chip bedding at 21 ± 2 °C and 36 ± 6% relative humidity under a 12-h light/dark cycle (lights on at 08:00), given free access to distilled water and irradiated diet. Prior to study initiation, all animals were acclimated for 1 week so as to recovery from transport stress. The general health status of the mice was evaluated by measuring weight gain. The polypropylene animal cages (M3 cage 32 × 20 × 13 cm, Suzhou, China) and accessory equipment including feeders and watering devices were washed and autoclaved regularly to keep them clean and free from contamination before use. The irradiated diet, which were nutritionally consistent with the national standard GB 14924.3–2010 “Laboratory animals - Nutrients for formula feeds”, was obtained from the Double Lion Experimental Animal Feed Technology Co., LTD (Suzhou, China). Results of nutrition analysis are shown in Additional file 1: Table S1.

After acclimation as described above, the mice weighing 21.68 ± 1.29 g (mean ± SD) were individually housed and randomly grouped into ad libitum control group or intermittent fasting groups. To study the effect of different fasting regimens on food intake and gut microbiota, the mice in fasting group were then divided into three sub-groups according to the duration of daily fasting: 12 h, 16 h and 20 h (*n* = 15 per group). Sample size was determined based on previous studies on diet-microbiota interactions using mouse model [31, 32]. Fasting was chose to be performed at night due to the opposite circadian rhythms between mice and humans. All groups of mice were fed at 8:30 am every day. Fasting was begun in the afternoon. After 30 days of intermittent fasting, fasting was stopped and the mice were fed ad libitum for an extended 1 month. Control mice had ad libitum access to food and water around the clock during the study. Any individual mouse that died of unknown causes before termination of the experiment was excluded from the study. The animal cages and the bedding used in the cages ware changed every 10 days to keep the animals dry and clean. Daily food consumption of each individual mouse was calculated by subtracting the weight of leftover food from the total amount of food given. The fecal sample for each mouse was independently collected on day 30 and day 60, and stored in − 80 °C freezer until use.

After the study, all mice were killed by cervical dislocation and subsequently treated as non-hazardous waste. Animal care was performed according to the Animal Ethics Procedures and Guidelines of the People’s Republic of China, and the experimental protocol was approved by the local committee on animal use and protection, Northwest Minzu University.

### Next-generation sequencing

Fecal DNA extraction and next-generation sequencing of 16S ribosomal RNA gene amplicons were performed by NoveGene, as reported elsewhere [33]. Briefly, The fecal DNA was extracted by a modification of the cetyltrimethylammonium bromide method. The V3–4 region of the bacterial 16S rRNA gene was amplified using 341F/806R primers [34]. PCR amplification was carried out in a reaction mixture containing Phusion® High-Fidelity PCR Master Mix (New England Biolabs, Ipswich, MA, USA). A DNA library for next-generation sequencing was prepared using Ion Plus Fragment Library Kit 48 rxns (Thermo Scientific) following manufacturer’s instruction. Finally, the library was sequenced on an Illumina platform.

Paired-end reads (250 bp) were assigned to each sample by a unique barcode, after which the barcode and primer sequence were removed. Merged reads were quality checked by using split-libraries-fastq.py in QIIME ver 1.9.1 [35, 36]. Using the UCHIME algorithm, the reads were compared with the reference database (SILVA database) to remove chimera sequences. Effective sequences were analyzed with the Uparse software and those with ≥97% similarity were assigned to the same OTU [37]. Representative sequences were classified against the SILVA (v123) reference taxonomy using a negative Bayesian classifier implemented within mothur [38, 39]. Finally, a rarefied feature table was created at one depth of sequence per sample, and all of the downstream analyses were performed with this rarified OTU (operational taxonomic unit) table.

Alpha diversity indices (Shannon diversity and Simpson index) were calculated using alpha-diversity.py in QIIME. Beta diversity was computed using Bray-Curtis distance metrics. Principal Co-ordinates Analysis (PCoA) of Bray-Curtis distance was performed using the “vegan” package in R programming language [40]. Multivariate data analysis methods of Adonis (nonparametric manova) and ANOSIM (analysis of similarities) were used to identify whether the daily fasting duration had an effect on the microbial communities. To identify bacterial taxa whose sequences were differentially abundant between groups, LEfSe (linear discriminant analysis (LDA) coupled with effect size measurements) analysis was applied (http://huttenhower.sph.harvard.edu/galaxy).

### Data analysis

The difference in food intake, body weight, and alpha diversity indices of mouse gut microbiota, was determined by one-way ANOVA, followed by Tukey’s post hoc test for multiple comparisons (GraphPad Prism 8.0, San Diego, CA, USA). Pearson correlation between fasting hours and food intake was calculated using the cor.test function in R. Differences with a *p* - value less than 0.05 was considered to be statistically significant.

## Supplementary information

**Additional file 1: Table S1.** Nutritional analysis of the experimental diet.

**Additional file 2: Figure S1.** The relative abundances of the genus *Akkermansia* in the individual animals in the 16 h fasting group and CTR at day 30.

## Data Availability

The datasets generated and analysed during the current study are available in NCBI’s Sequence Read Archive (SRA) repository under the BioProject ID PRJNA592777 (https://www.ncbi.nlm.nih.gov/bioproject/592777).

## Abbreviations

hrs: Hours
CTR: Control
ANOSIM: Analysis of similarities
Adonis: A multivariate ANOVA based on similarity tests
PCoA: Principal coordinates analysis
LDA: Liner Discriminate Analysis
LEfSe: Liner Discriminate Analysis (LDA) coupled with effect size measurements
OTUs: Operational taxonomic units
OTU: Operational taxonomic unit
