# Supplementary material for: The effects of daily fasting hours on shaping gut microbiota in mice
Source: BMC Microbiol. 2020 Mar 24;20:65. doi: 10.1186/s12866-020-01754-2 (PMC7092480; doi:10.1186/s12866-020-01754-2)
Supplement: Supplementary file 1 — Additional file 1: Table S1. Nutritional analysis of the experimental diet. [file 12866_2020_1754_MOESM1_ESM.docx]

Table S1. Nutritional analysis of the experimental diet.

| Experimental diet | g/kg of diet |
| --- | --- |
| Composition |  |
| Moisture | 100 |
| Crude protein | 200 |
| Crude fat | 40 |
| Crude fiber | 50 |
| Ash | 80 |
| Calcium | 10-18 |
| Total phosphorus | 6-12 |
| Calcium: total phosphorus | 1.2:1-1.7:1 |
| Amino acids |  |
| Lysine | 13.2 |
| Methionine & Cystine | 7.8 |
| Arginine | 11.0 |
| Histidine | 5.5 |
| Tryptophan | 2.5 |
| Phenylalanine & Tyrosine | 13.0 |
| Threonine | 8.8 |
| Leucine | 17.6 |
| Isoleucine | 10.3 |
| Valine | 11.7 |
| Vitamins |  |
| Vitamin A (IU/kg) | 14000 |
| Vitamin D (IU/kg) | 1500 |
| Vitamin E (IU/kg) | 120 |
| Vitamin K | 0.005 |
| Vitamin B1 | 0.013 |
| Vitamin B2 | 0.012 |
| Vitamin B6 | 0.012 |
| Niacin | 0.06 |
| Pantothenic acid | 0.024 |
| Folic acid | 0.006 |
| Biotin | 2.0E-4 |
| Vitamin B12 | 2.2E-5 |
| Choline | 1.25 |
| Vitamin C | 0 |
| Minerals |  |
| Magnesium | 2.0 |
| Potassium | 5 |
| Sodium | 2.0 |
| Iron | 0.12 |
| Manganese | 0.075 |
| Copper | 0.01 |
| Zinc | 0.03 |
| Iodine | 5.0E-4 |
| Selenium | (1.0-2.0)E-4 |
