# Supplementary figures and images for: The effects of daily fasting hours on shaping gut microbiota in mice
Source: BMC Microbiol. 2020 Mar 24;20:65. doi: 10.1186/s12866-020-01754-2 (PMC7092480; doi:10.1186/s12866-020-01754-2)

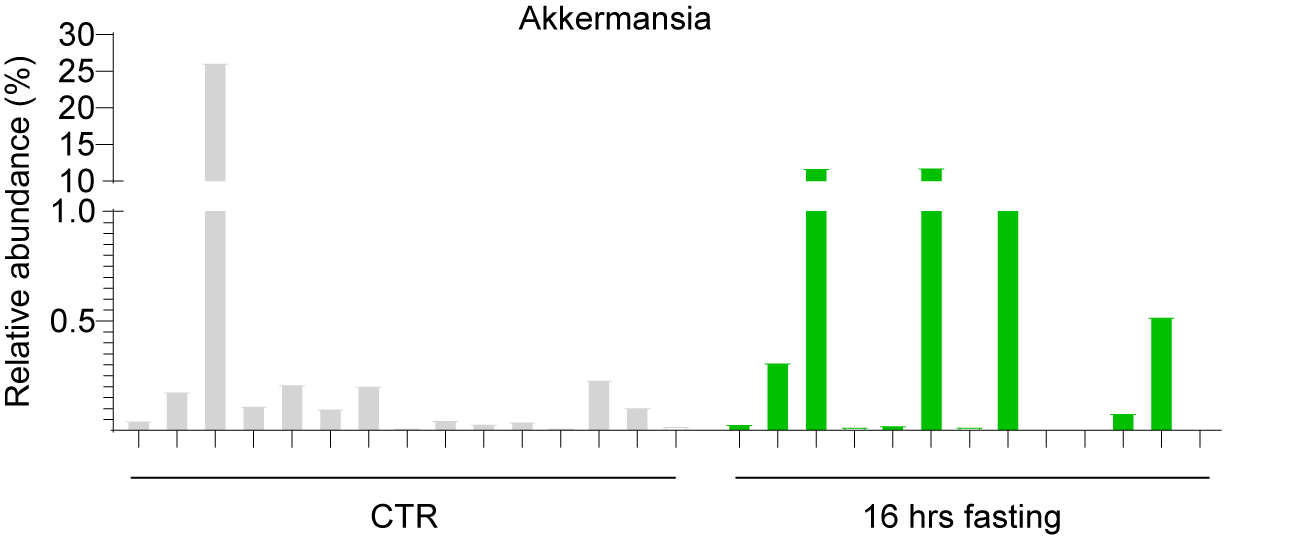

Supplement: Supplementary file 2 — Additional file 2: Figure S1. The relative abundances of the genus Akkermansia in the individual animals in the 16 h fasting group and CTR at day 30. [file 12866_2020_1754_MOESM2_ESM.tif]
